# Supplementary material for: Sensitive detection and quantification of SARS-CoV-2 in saliva
Source: Sci Rep. 2021 Jun 14;11:12425. doi: 10.1038/s41598-021-91835-7 (PMC8203799; doi:10.1038/s41598-021-91835-7)
Supplement: Supplementary file 13 — Supplementary Legends. [file 41598_2021_91835_MOESM13_ESM.docx]

**Supplementary materials**

**Supplementary Figure 1:** RT-PCR calibration curves for N1 and N2 targets. Serial dilution of N1 and N2 standards showed calibration curves with high concordance and linearity. The RT-PCR efficiency for each target are greater than 99.25 %. The lowest standard concentration used (i.e., 0.1CN/µl, CN: copy number) can be easily discriminated from negative template control (whose Ct value was 45). The technical limit of the assay is 1 CN of target in a reaction mixture of 20 µL, with corresponding Ct cycles (according to our calculated slope and intercept) are 38.2 and 38.6 cycles for N1 and N2 respectfully. We used the CDC guided Ct threshold (i.e., <40) to call a sample positive (n=3, the error bars: Standard Error). ΔCt values were calculated as follows: mean Ct value of no template control (NTC) minus mean Ct value of target gene.

**Supplementary Figure 2:** ddPCR calibration curves for N1 and N2 targets. Serial dilution of N1 and N2 standards showed calibration curves with high concordance and linearity. The limit of detection (LOD) for N1 and N2 targets are 0.06 and 0.21 CN/µl, respectively (n=2, the error bars: Standard Error).

**Supplementary Figure 3:** ddPCR and RT-PCR results from 26 clinically collected nasopharyngeal samples (8 positive and 18 negative by the EUA approved Xpert assay). (Left) Measurements of the RnaseP target quantified by ddPCR and RT-PCR display high correlation (Slope: 1.4 and R^2^:1.0) (n=1). (Right) The positive and negative results were compared with those of CML and showed a good concordance. Both ddPCR and RT-PCR have properly confirmed all positive results of CML. In addition, 13 and 11 CML negative samples were caught as positive by RT-PCR and ddPCR respectively.

**Supplementary Figure 4:** Trizol RNA Extraction Protocol (TRE) was compared with two commercial kits (i.e., QIAmp MinElute Virus DNA/RNA Spin Kit (QVDRK), QIAmp Viral RNA Mini Kit (QVK)). In this experiment, a saliva sample was aliquot into 9 vials, and each three vials were extracted by the same kit/protocol. All extracts were analyzed at the same time by RT-PCR and ddPCR to quantify RnaseP target (n=3, Error bars: Standard Deviation).

**Supplementary Figure 5**: (a) RT-PCR assay validation with positive SARS-CoV-2 RNA standard dilution curve and no-template control (NTC) on all 10 plates. Standard dilution curves on three selected plates are shown in (b).

**Supplementary Figure 6:** (a) ddPCR assay validation with positive SARS-CoV-2 RNA standard and no-template control (NTC) on all 17 plates, in terms of copy number per µL. (b) ddPCR assay validation with positive SARS-CoV-2 RNA standard and no-template control (NTC) on all 17 plates, in terms of positive droplet number.

**Supplementary Figure 7:** Histograms demonstrate ddPCR analysis of the specimens collected from COVID-19 negative and asymptomatic patients (n=45). Nasal swab and saliva samples were collected from symptom-free patients undergoing pre-operation screening (N = 45) and measured with digital PCR to establish an empirical threshold. The y-axis unit for all subplots are occurrences; the x-axis unit for subplots are either copy number per µL (CN/µL), or total number of positive droplets in the reaction (“pos droplet”). The thresholds were calculated as: mean+2*SD (SD: standard deviation).

**Supplementary Figure 8**: Reproducibility analysis of ddPCR assay for saliva specimens. The same extracted RNA samples (stored in -80 ºC) have been re-analyzed after ~5 months. The results indicate a high level of reproducibility. (a) The comparison of N1 target measurements of first and second analysis in terms of positive droplet number (left) and CN/µL (right); (b) The comparison of target N2 target measurements of first and second analysis in terms of positive droplet number (left) and CN/µL (right).

**Supplementary Table 1.** Comparative analysis of the EUA Xpert test performed at the microbiology laboratory, EUA TaqPath COVID-19 Combo Kit, and research laboratory-based RT-PCR/ddPCR approaches for detection of SARS-CoV-2 in pooled and diluted NP samples. Each sample was tested in duplicates.

**Supplementary Table 2**: ddPCR thresholds for analysis of NS and saliva samples. Thresholds (in term of both CN/µL and total positive droplet numbers) were determined by measuring NS and saliva samples collected from COVID-19 negative patients tested at the curbside as part of their pre-operative screening (also see **Supplementary Figure 6** and **7**).

**Supplementary Table 3:** List of 46 saliva samples selected for re-analysis by ddPCR.

**Supplementary Table 4:** Viral load in samples (CN/1 ml).
